# *Toxoplasma* Hypervirulence in the Rat Model Parallels Human Infection and Is Modulated by the *Toxo1* Locus

**DOI:** 10.3389/fcimb.2019.00134

**Published:** 2019-05-01

**Authors:** Corinne Loeuillet, Anais Mondon, Salima Kamche, Véronique Curri, Jean Boutonnat, Pierre Cavaillès, Marie-France Cesbron-Delauw

**Affiliations:** ^1^BNI Team, Grenoble Alpes, CNRS, Grenoble INP, TIMC-IMAG, Grenoble, France; ^2^Therex Team, Grenoble Alpes, CNRS, Grenoble INP, TIMC-IMAG, Grenoble, France; ^3^Unit of Anatomopathology, Institute of Biology and Pathology, Grenoble Alpes Hospital, Grenoble, France

**Keywords:** *Toxoplasma*, virulence, GUY008-ABE, *Toxo1*, resistance, rat model

## Abstract

Toxoplasmosis is considered as an opportunistic parasitic disease. If post-natally acquired in children or adults, it may pass unnoticed, at least with strains of European origin. However, in the wild biotopes especially in South America, *Toxoplasma gondii* strains display a greater genetic diversity, which correlates to higher virulence for humans, particularly along the Amazon River and its tributaries. In French Guiana, several atypical strains have been associated with severe clinical forms: ocular toxoplasmosis and acute respiratory distress syndrome both of which can result in death. Among these, the GUY008-ABE strain was responsible for an epidemic of severe disseminated toxoplasmosis in Suriname, which led to the death of one immunocompetent individual. To better understand the mechanism underlying the hypervirulence of the GUY008-ABE strain, we have tested the rat model which compared to the mouse, better reflects the immune resistance of humans to *Toxoplasma* infection. Here we compare the outcome of toxoplasmosis in F344 rats infected either by the GUY008-ABE strain or the type II Prugniaud strain. We show that the GUY008-ABE strain displays a higher virulence phenotype leading to the death of all infected rats observed in this study. GUY008-ABE infection was characterized by an increase of the parasite load in several organs, especially the heart and lung, and was mainly associated with severe histological changes in lungs. Moreover, correlating with its hypervirulence trait, the GUY008-ABE strain was able to form cysts in the LEW rat model otherwise known to be refractory to infection by other *Toxoplasma* strains. Together, these results show that the rat is a discriminating experimental model to study *Toxoplasma* virulence factors relevant to the pathogenesis of human infection and that the degree of virulence is linked to the *Toxo1* locus.

## Introduction

*Toxoplasma gondii* is one of the most widespread parasites in the world. It is an obligate intracellular parasite which can infect all warm-blooded animals including humans (Hill and Dubey, 2002). Infection is acquired by ingesting either tissue cysts (in undercooked meat) or oocysts excreted in the environment by felidae (cats in particular) (Hill and Dubey, 2002).

The *T. gondii* population structure is clonal with most of the isolates found in Europe and North America belonging to three major haplotypes (I, II, and III), and recently the newly described haplotype 12 which is present in North America (Khan et al., 2011). In these countries, the type II is responsible of the majority of human cases of toxoplasmosis. While the infection may lead to severe or life-threatening illness in immunocompromised patients (Porter and Sande, 1992) and in congenitally infected newborns (McAuley et al., 1994), it is typically asymptomatic in healthy individuals, present as dormant cysts in brain and heart muscle (Tenter et al., 2000).

However, the genomic diversity of *T. gondii* strains is far higher in other parts of the world, especially in tropical South America (Shwab et al., 2014; Lorenzi et al., 2016) and is associated with a higher pathogenicity in healthy individuals (Dubey et al., 2012; Carneiro et al., 2013). In the French Guiana, some strains like the GUY008-ABE, have provoked outbreaks with severe forms including acute respiratory distress syndrome, sometimes with a lethal outcome (Demar et al., 2012). The discovery of strains highly virulent for humans has enlarged the possibility of finding new virulence factors of *T. gondii* and the corresponding interacting-host pathways (Melo et al., 2013).

To explore complex virulence traits, we require animal models representative of human infection for experimentation. One recent study described the infection outcome of seven French Guiana *Toxoplasma* strains in CD1 mice and showed a correlation between the variability of the mouse chromosome 1a (chr1a) and mice lethality (Khan et al., 2014). However, while mice are among the most highly susceptible species to toxoplasmosis (Zenner et al., 1998), the rat model by its natural resistance to *Toxoplasma* infection is more relevant for human toxoplasmosis and opportunity's better model for understanding mechanisms controlling human infection (Dubey and Frenkel, 1998). Most rat strains develop asymptomatic toxoplasmosis with the persistence of cysts in brain and muscles. Other strains of rat like the Lewis (LEW) are refractory and efficiently prevent the parasite dissemination into the body. This innate resistance response to *Toxoplasma* infection is mainly controlled by a single locus called *Toxo1* located on the rat chromosome 10 (Sergent et al., 2005; Cavaillès et al., 2006). In humans, the *Toxo1* locus is associated with the susceptibility to congenital toxoplasmosis (Witola et al., 2011, 2014).

In this work, we examined the outcome of *Toxoplasma* infection in rats infected with the GUY008-ABE strain responsible for an epidemic episode in Suriname with severe disseminated, even lethal toxoplasmosis (Demar et al., 2007). By contrast to the type II Prugniaud strain, the GUY008-ABE strain displayed high virulence in infected F344 rats leading to the death of the animals. We also showed that in the LEW rat, the GUY008-ABE strain could establish a silent chronic infection, indicating that the *Toxo1* locus may modulate virulence without blocking parasite dissemination.

## Materiels and methods

### *Toxoplasma* Strains and Culture

The highly virulent Guiana GUY008-ABE strain was characterized and compared to Prugniaud type II strain. All the parasite strains were maintained by serial passages in primary human foreskin fibroblasts (HFF) at 37°C with 5% CO_2_ in a humidified atmosphere. HFFs were grown in Dulbecco's modified Eagle medium (DMEM, GibcoBRL) supplemented with 10% FCS, 2 mM glutamine, 100 U/ml penicillin and 100 U/ml streptomycin (Invitrogen).

### Ethics Statement

Breeding and experimental procedures were carried out in accordance with national and international laws for laboratory animal welfare and experimentation (EEC Council Directive 2010/63/EU, September 2010). Experiments were performed under the supervision of C.L. (agreement 38 10 38) in the “Plateforme de Haute Technologie Animale (PHTA)” animal care facility (agreement C3851610006 delivered by the “Direction Départementale de la Protection des Populations”) and were approved by the ethics committee of the PHTA and by the French government (APAFIS#7617-2016111710364203 v3).

### Rat Strains, Inoculation and Disease Monitoring

Lewis (LEW) and Fischer (F344) rats were purchased from Janvier and Charles Rivers laboratories, respectively. Congenic LEW.BN.c10-F strains were bred in PHTA platform. All these rats were housed under specific pathogen-free conditions.

To study the acute phase of infection, F344 rats were anesthetized with isofluorane (Abbot Laboratories) and subsequently inoculated intraperitoneally (i.p.) with 10^7^ tachyzoites of the GUY008-ABE or the Prugniaud strains. At days 0, 4, and 14, rat's weights were checked and rat survival monitored. Then, to characterize parasite dissemination, animals were infected and organs were sampled at days 4 and 14. Heart, lungs, mesenteric lymph nodes, brain, liver and spleen were separated in two parts: one part was fixed in formol for subsequent histological study, the other was placed directly in liquid nitrogen and then preserved at −80°C before DNA extraction and parasite load determination by quantitative PCR.

To look for influence of the *Toxo1* locus on infection outcome, resistant LEW and susceptible congenic line LEW.BN.c10-F rats were i.p. inoculated with 10^7^ tachyzoites of each strain (GUY008-ABE and Prugniaud). Then, 2 months post-infection, quantification of brain cysts was assessed as described previously (Aldebert et al., 2011; Cavailles et al., 2014).

### DNA Extraction and Parasite Load Determination

DNA was extracted from each organ sample (20 mg) by using the Purelink Genomic DNA mini kit (Invitrogen) and following the manufacturers' recommendations. A final elution of 50 μL for each sample was performed.

The parasite load in heart, lungs, brain, liver and spleen was estimated by quantitative PCR on a Step One Plus real time PCR system apparatus (Applied Biosystems).

Quantitative standard curve was obtained by 8 fold-dilutions ranging from 5,26 × 10^5^-5.26 × 10^−2^ tachyzoïtes.

DNA samples were analyzed using 300 nM of primers (Eurogentec) targeting the 529 bp repeated sequence: 5′-GCGTCGTCTCGTCTAGATCG-3′ for the forward and 5′-AGGAGAGATATCAGGACTGTAG-3′ for the reverse, 5 μL of Fast SyBr-Green (Life Technologies) and 1 μL of 1/50 diluted DNA, qsp H20 up to 10 μL. Forty PCR cycles were: 95°C, 20 s; 40 × (95°C, 3 s; 62°C, 30 s; 72°C, 15 s), finally a melting curve was performed.

For each sample, the final value of the parasite load was given by the following equation: Parasite load = (Nx50xorgans' weight (mg))/(samples' weight (mg)), with N is the number of parasites obtained by the qPCR.

### Tissue Histological Analysis

The brains, lungs, spleens, livers, hearts samples were collected, fixed in 10% buffered formalin and processed routinely for paraffin embedding and sectioning. Tissue sections with 4 μm thickness (40 μm distance between sections) of each organ were cut with microtome (Leica, RM2245) and mounted on slides for histopathological study. Tissue sections were stained with Haematoxylin and Eosin and they were observed under light microscope. These manipulations have been made on the histological platform of Jean Roget Institute, La Tronche.

### Parasites Proliferation in Rat Peritoneal Macrophages

Rat resident peritoneal cells were obtained by injection of sterile PBS into the peritoneal cavity. Collected cells were centrifuged, resuspended in Serum-Free Medium (SFM, Life Technologies, Inc) and counted. Macrophages were obtained by adhering cells for 1 h at 37°C and 5% CO_2_. After 1 h, non-adherent cells were removed by gentle washing with SFM and SFM medium supplemented with 20% of L929-conditioned media added. After 24 h, parasites were added to macrophages at a ratio of 3:1 for 1 h. After washing to eliminate extracellular parasites, cells were cultured for 40 h in the presence of [3H] uracil (5 μCi per well, Ci = 37 GBq) as previously described (Pfefferkorn and Pfefferkorn, 1977). Monolayers were washed three times in PBS, disrupted with 500 μl of lysis/scintillation solution (Optiphase Supermix, Perkin Elmer) and radioactivity measured by liquid scintillation counting using a Wallac MicroBeta TriLux (Perkin Elmer).

## Results

### The Infection of F344 Rats by the French Guianan *Toxoplasma* Strain GUY008-ABE Is Lethal

**Figure 1:**
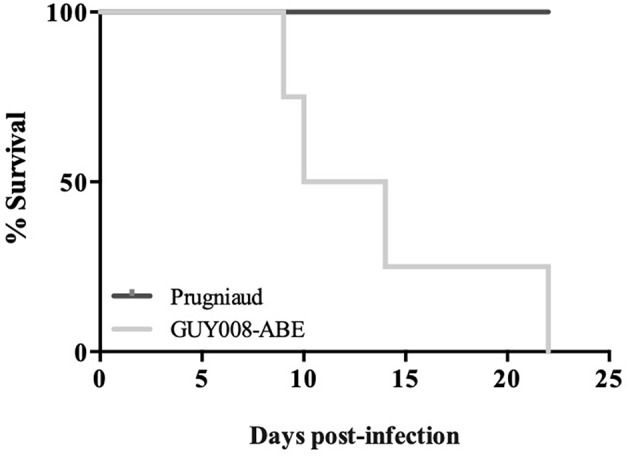
Hypervirulence of the GUY008-ABE strain in the susceptible F344 rats. Rats were intraperitoneally infected with 10^7^ tachyzoites of type II Prugniaud (*n* = 5) or Guy008-ABE (*n* = 6) strains and daily monitored.

We first analyzed rat survival following intraperitoneal inoculation of F344 rats by the GUY008-ABE strain or the avirulent type II Prugniaud strain. As expected, F344 rats infected with the Prugniaud strain, did not present clinical signs and all animals survived until 2 months post-infection (Figure 1). By contrast, while no clinical signs were noticeable in rats infected by the GUY008-ABE strain during the first seven days-post infection, a rapid decline of their health status was observed after 10 days with symptoms including respiratory distress, asthenia, emaciation and orbital hemorrhage. All the GUY008-ABE-infected F344 rats died within 22 days post-infection (Figure 1). These results demonstrate that the F344 rat model discriminates between virulent and avirulent *Toxoplasma* strains.

### GUY008-ABE Infection Is Responsible for Rat Weight Reduction as Well as Organomegalies

**Figure 2:**
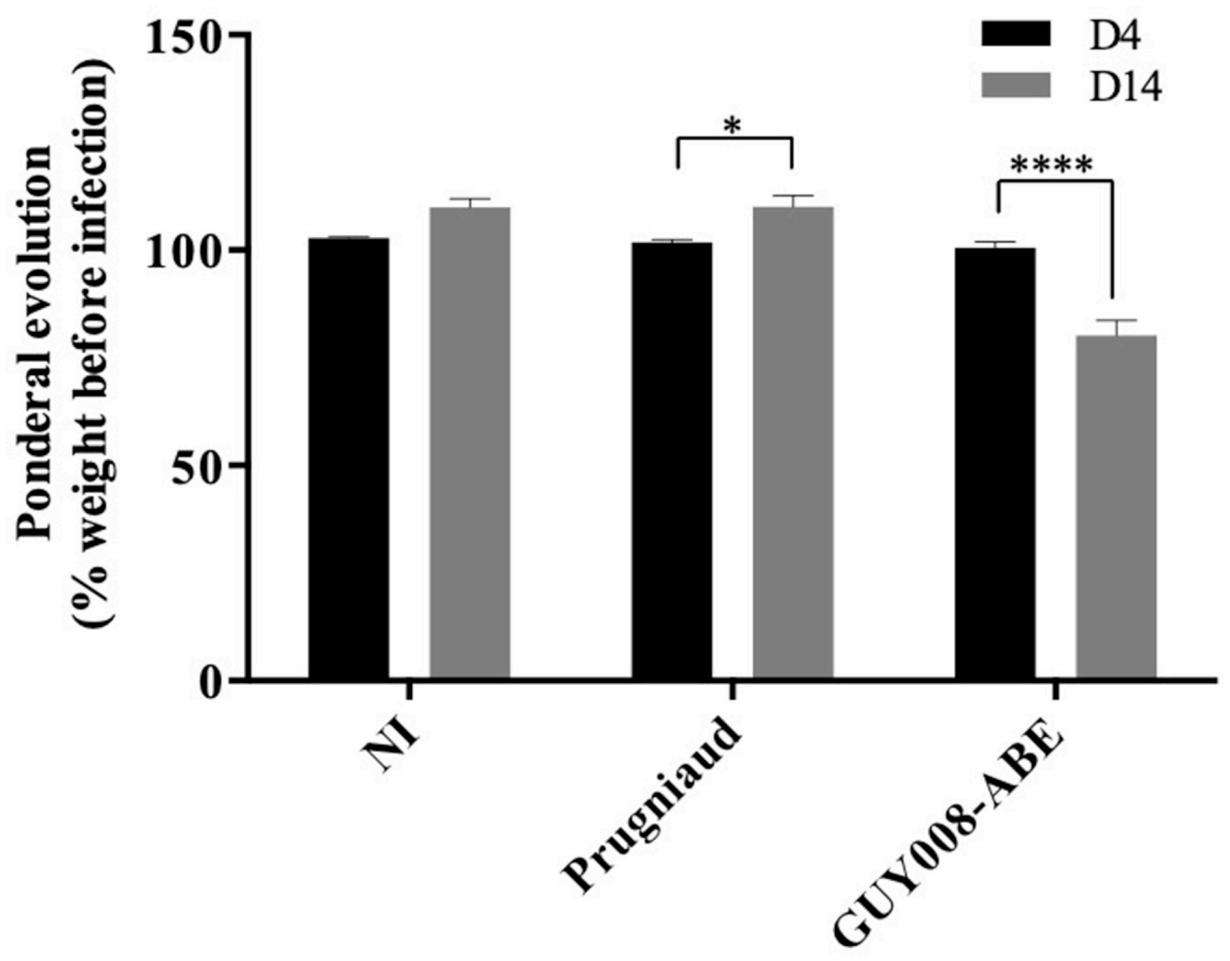
Ponderal evolution of F344 rats after *Toxoplasma gondii* infection. Rats were intraperitoneally infected with 10^7^ tachyzoites of type II Prugniaud (*n* = 8) or GUY008-ABE (*n* = 8) strains. Un-infected (NI) rats were used as controls (*n* = 5). Animals were weighted at days 4 and 14 post-infection. Percentages of weight regarding animal weight before infection were calculated and means ± SEM represented. Values were submitted to 2way ANOVA analysis followed by a Tukey's multiple comparisons test (*p < 0.05, ****p < 0.0005).

We found that GUY008-ABE infection resulted in deaths of 75% of the rats after 15 days post-infection (Figure 1). In order to define more precisely the pathological processes leading to the animal death, the F344 rats were infected intraperitoneally and sacrificed at day 4 (D4) or day 14 (D14) post-infection. Between D4 and D14, un-infected (NI) or Prugniaud-infected rats gained 10% of their weight load (Figure 2, *p* < 0.05 for Prugniaud) whereas GUY008-ABE-infected rats lost 20% of their weight load (Figure 2, *p* < 0.0005).

**Figure 3:**
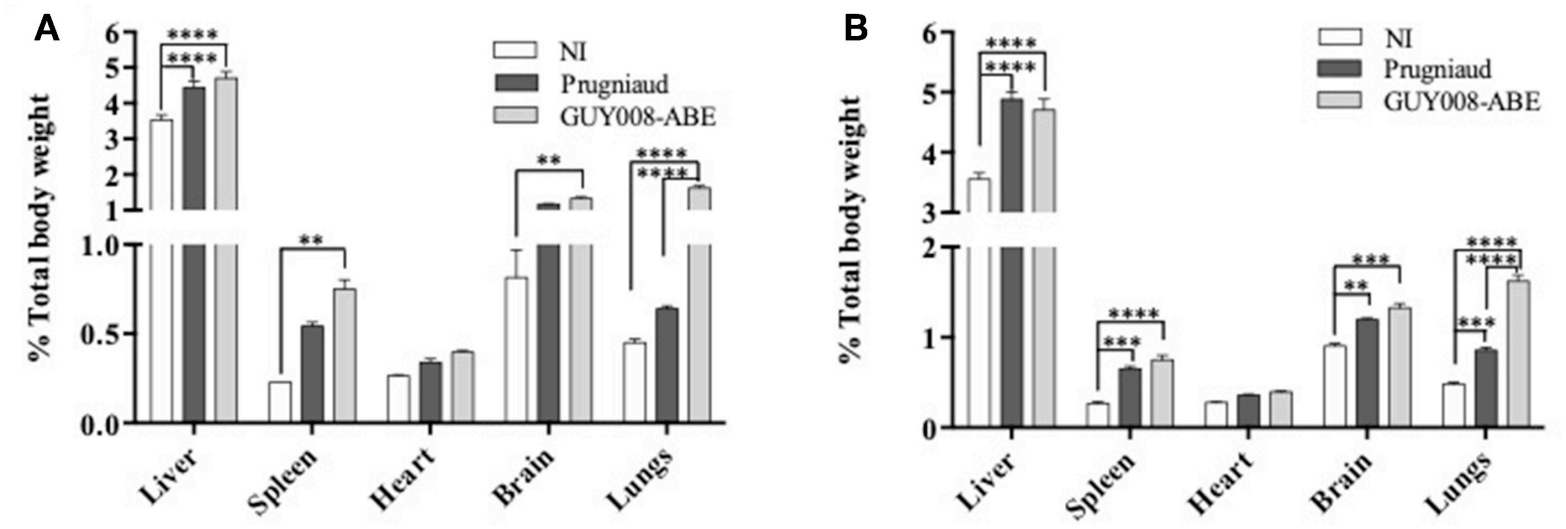
Ponderal indexes of rat organs following *Toxoplasma gondii* infection. Rats were intraperitoneally infected with 10^7^ tachyzoites of type II Prugniaud (*n* = 8) or Guy008-ABE (*n* = 8) strains. Un-infected (NI) rats were used as controls (*n* = 3). Animals were sacrificed at days 4 **(A)** or 14 post-infection **(B)**. Percentages of organs weight regarding total animal weight were calculated and mean ± SEM represented. Values were submitted to 2way ANOVA analysis followed by a Tukey's multiple comparisons test (**p < 0.005, ***p < 0.0005, ****p < 0.00005).

Guianese toxoplasmosis is known in humans to be associated with respiratory distress, hepato- and spleno-megalies (Carme et al., 2002), we decided to study the weight evolution of liver, spleen, lung, and heart after infection by Prugniaud or GUY008-ABE strains (Figure 3). Because of cyst formation in the brain, we also analyzed the weight of this organ. Since the GUY008-ABE-infected rats presented a global weight loss, the weights of organs were normalized to the rat weight before infection. Compared to NI rats and independent of the parasite strain, weight increase was observed for all organs of infected rats at both days 4 and 14 (Figures 3A,B, respectively) after infection. Additionally, inspection of GUY008-ABE-infected organs at day 14 showed a highly significant weight increase for the lungs compared to Prugniaud infected lungs. This lung swelling is therefore a signature of the GUY008-ABE hypervirulence.

### The Parasite Load Is More Important in the GUY008-ABE-Infected Rat and Is Associated With Severe Histological Changes

**Figure 4:**
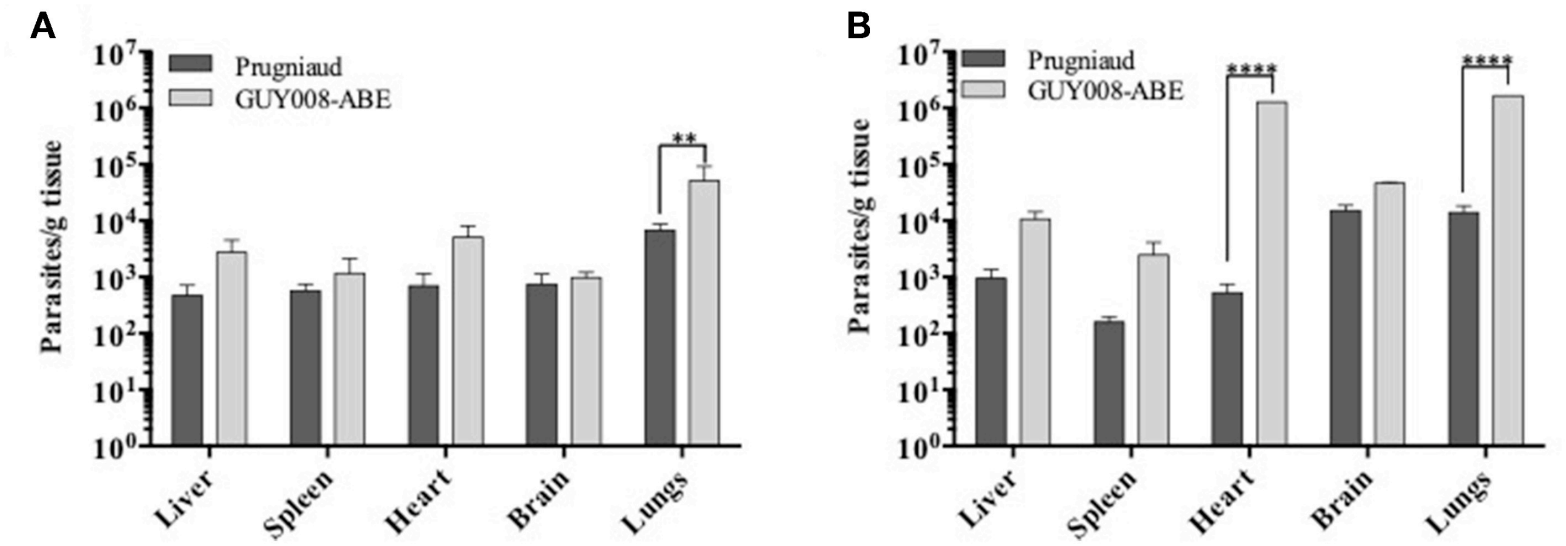
Parasites load of rat organs following *Toxoplasma gondii* infection. Rats were intraperitoneally infected with 10^7^ tachyzoites of type II Prugniaud (*n* = 8) or GUY008-ABE (*n* = 8) strains. Animals were sacrificed at days 4 **(A)** or 14 post-infection **(B)**. Parasite loads were expressed as parasites per tissue gram and mean ± SEM represented. Values were submitted to 2way ANOVA analysis followed by a Tukey's multiple comparisons test (**p < 0.005, ****p < 0.00005).

We further compared the parasite load at days 4 and 14 after infection. Parasites were detected within all the tested organs of both Prugniaud- and GUY008-ABE-infected rats (Figure 4). At day 4 post-infection, significant difference was noticed between GUY008-ABE and Prugniaud infections for the lungs only (Figure 4A). At day 14 post-infection, a high increase of the parasite load was observed in both the hearts (2,419 times increased by comparison to day 4) and lungs (117 times increased by comparison to day 4), specifically in GUY008-ABE-infected rats. The higher parasite load in heart and lungs is thus associated with the strain virulence.

**Figure 5:**
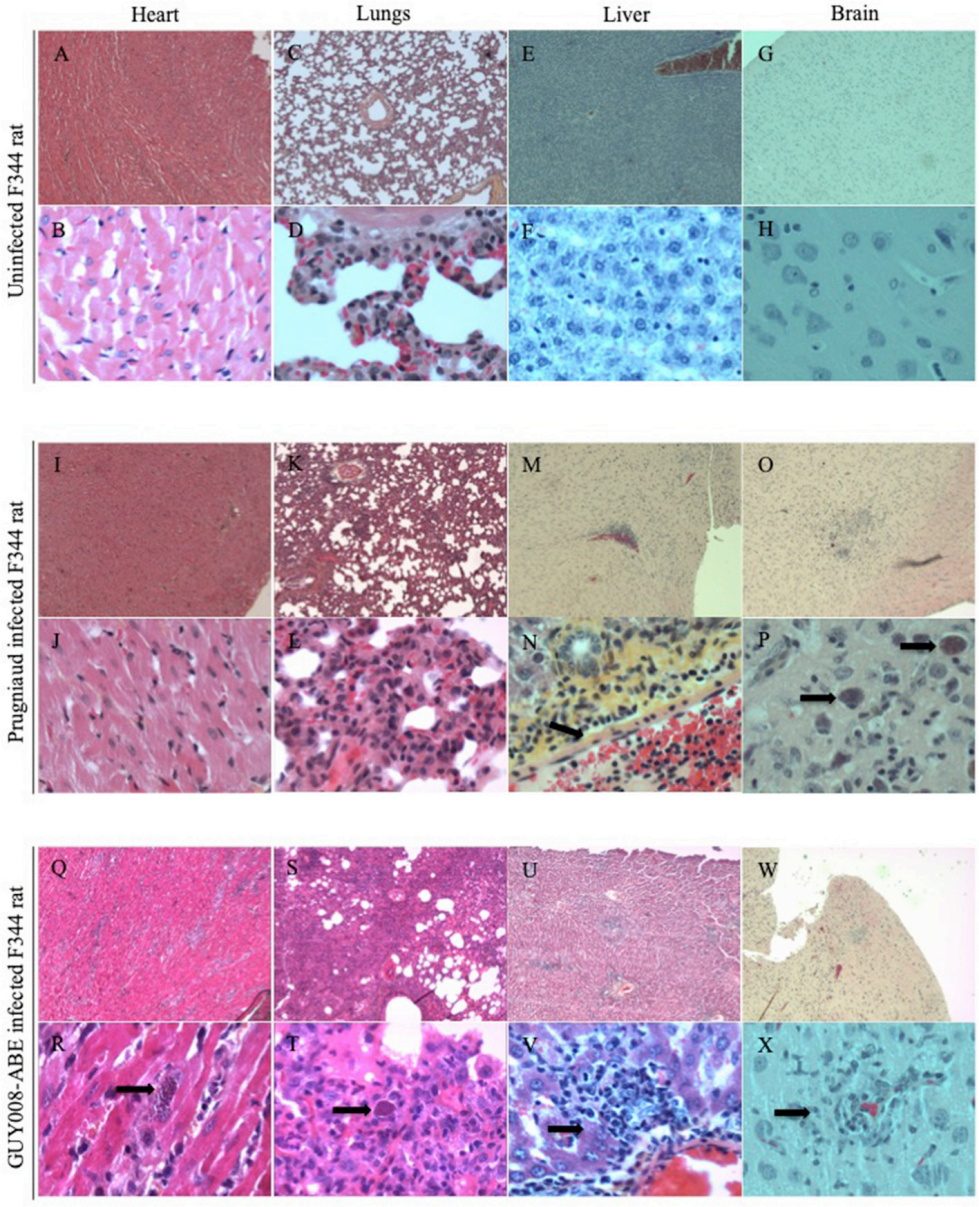
GUY008-ABE induced heart and lungs tissues reorganization. Rats were intraperitoneally infected with 10^7^ tachyzoites of type II Prugniaud or GUY008-ABE strains and organs histological analysis performed at day14 post-infection with an haematoxilin and eosin staining. **(A–H)** Represent the un-infected F344 rat, **(I–P)** the Prugniaud-infected rat and **(Q–X)** the GUY008-ABE-infected rat. **(A,B,I,J,Q,R)** Correspond to heart histological images, **(C,D,K,L,S,T)** to lungs images, **(E,F,M,N,U,V)** to liver images and **(G,H,O,P,W,X)** to brain images. Magnifications were x4 for panels **(A,C,E,G,I,K,M,O,Q,S,U,W)** and x40 for **(B,D,F,H,I,L,N,P,R,T,V,X)**. Arrows on **(N)** indicate the portal vein sinus; on **(P,R,T)**, parasite foci; on **(V)**, histiocytic infiltration and on **(X)** parasites inside macrophages.

Tissues observations were realized at days 4 and 14 post-infection in both infected rats (Figure 5). No tissue perturbations were observed at day 4 post-infection when comparison was done regarding the tissue of NI rats and whatever the parasite strain used (GUY008-ABE or Prugniaud) for rat infection (not shown). At day 14 post-infection, comparison to the NI rat (Figures 5A–H) revealed that for the Prugniaud-infected rats, there was no abnormality in heart (Figures 5I,J), mesenteric lymph nodes, lung (Figures 5K,L) or spleen. In the liver, a slight inflammation was observed surrounding the portal vein sinus (Figure 5N). Parasites were found in the brain macrophages (Figure 5P) and a perivascular lymphocyte infiltration was also observed. For the GUY008-ABE-infected rats, no histological change was detected in spleen, thymus, or mesenteric lymph nodes. However, damage could be seen in other organs. In the liver, we observed a reaction of lymphohistiocytic vessels in response to infection (Figure 5V) and the presence of parasite granuloma. In the heart, as in the brain, focal inflammatory reactions were detected as well as parasite granuloma (Figures 5R,X). The lungs are also greatly affected with a histiocytic inflammatory reaction and the presence of parasite foci (Figures 5S,T). These latter observations (inflammation and presence of parasites in both lungs and heart) highly correlated with the respiratory distress observed in GUY008-ABE-infected animals.

### Influence of the *Toxo1* Locus on Guianese Toxoplasmosis

**Figure 6:**
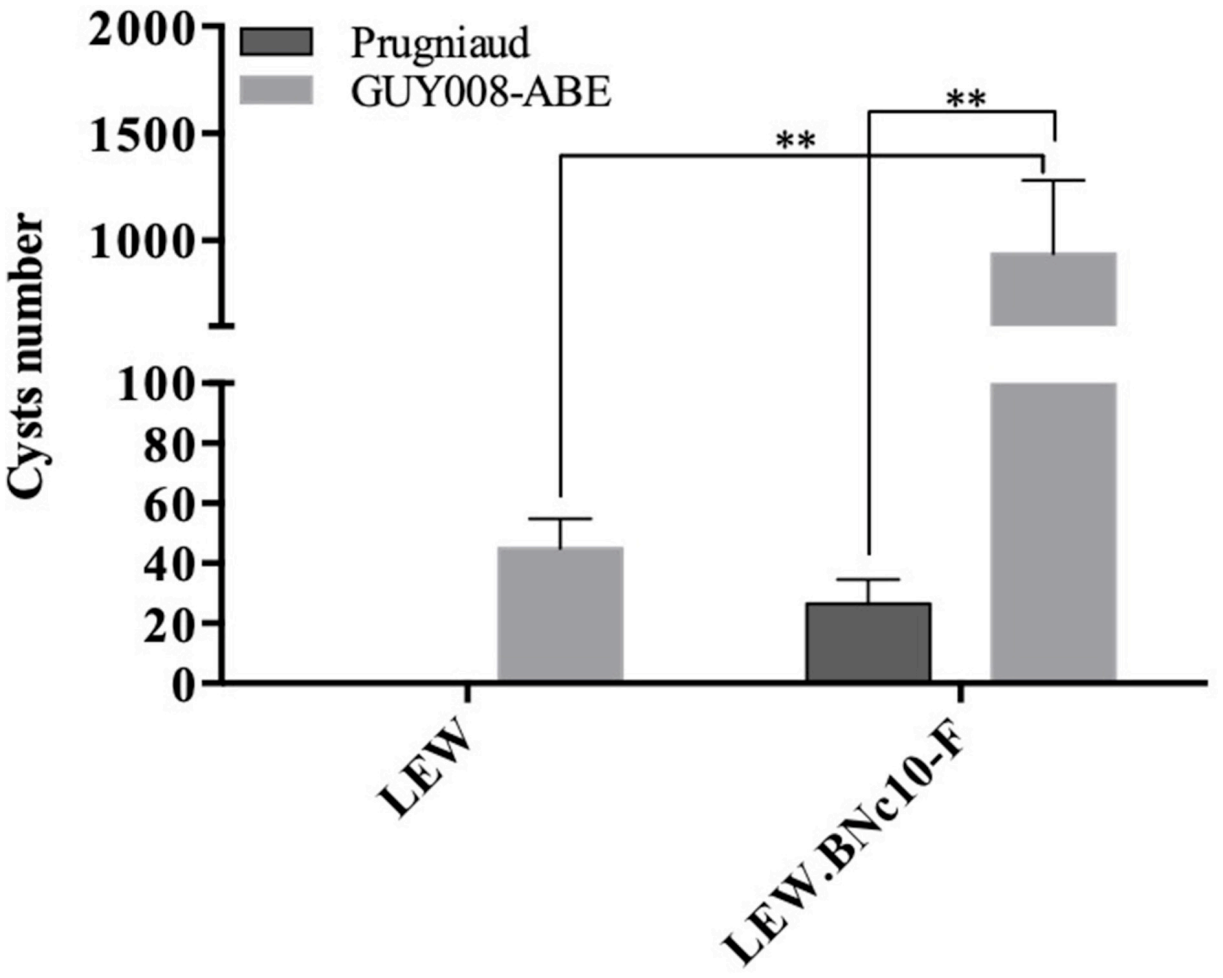
Influence of *Toxo1* on Guiana toxoplasmosis infection outcome. Rats were intraperitoneally infected with 10^7^ tachyzoites of type II Prugniaud (*n* = 6) or GUY008-ABE (*n* = 6) strains. Animals were sacrificed 2 months post-infection and presence of cerebral cysts assessed. Cyst values were submitted to 2-way ANOVA analysis followed by a Tukey's multiple comparisons test (**p < 0.005).

In order to analyze the involvement of the *Toxo1* locus in the control of Guianese toxoplasmosis, resistant LEW and susceptible LEW congenic rats with *Toxo1* from BN origin (LEW.BNc10-F) were intraperitoneally infected with the GUY008-ABE strain (Figure 6). As expected, no cyst was found in the brain of LEW rats infected with Prugniaud strain while cyst burden was found in the LEW.BNc10-F (Cavaillès et al., 2006). By contrast, GUY008-ABE-infected LEW rats displayed brain cysts (mean = 45) indicating that GUY008-ABE parasites were able to bypass *Toxo1*-mediated refractoriness. Moreover, in the susceptible LEW.BN.c10-F rats, the number of brain cysts was significantly higher (an average of 482 cysts per brain). These results demonstrated that the hypervirulence of GUY008-ABE strain is associated with its capacity to modulate the *Toxo1*-mediated resistance and conversely, that the *Toxo1* locus controls GUY008-ABE strain hypervirulence, in negatively impacting cyst burden.

### Parasite Proliferation in Peritoneal Macrophages

**Figure 7:**
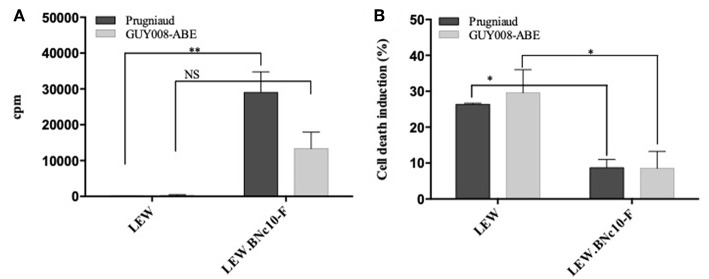
GUY008-ABE parasites do not proliferate in resistant LEW macrophages. **(A)** Peritoneal macrophages were infected (MOI 1:3) and parasites proliferation assessed by 3H uracil incorporation 40 h post-infection. Results were normalized according to the values obtained in non-infected macrophages. Means ± SEM of 3 independent experiments are represented. **(B)** Cell death was monitored by propidium iodide (PI, 5 μg/ml) uptake 4 h post-infection. Histograms represent the percentage of PI positive dying cells (results indicate the difference between infected and un-infected cells). Means ± SEM of 3 independent experiments are represented. Values were submitted to 2-way ANOVA analysis followed by a Tukey's multiple comparisons test (*p < 0.05; **p < 0.005). NS, non-significant.

The *Toxo1*-mediated *in vivo* resistance has been correlated to the *in vitro* inhibition of parasite proliferation in LEW peritoneal macrophages and to both macrophages and parasite death (Cavailles et al., 2014). Here, we have examined the capacity of GUY008-ABE parasites to proliferate either in resistant LEW or susceptible LEW.BNc10-F peritoneal macrophages by comparison to Prugniaud strain (Figure 7). We showed that Prugniaud and GUY008-ABE strains do not proliferate in LEW macrophages (Figure 7A) and this correlated with the death of infected cells (Figure 7B). These results reveal that for the GUY008-ABE strain, there is a discrepancy between the observed *in vitro* and *in vivo* phenotypes.

## Discussion

Successful pathogenicity depends on both host and parasite genotypes and environmental factors which can be controlled in experimental animal models. Using the F344 rat model of *Toxoplasma* infection, we showed here that the atypical GUY008-ABE strain, by contrast to the Prugniaud strain, exhibits a highly virulent phenotype in leading to the death of animals. In this model, the symptoms and pathophysiology of infection parallel that found in patients infected by the GUY008-ABE strain (Demar et al., 2007). We also demonstrated that the virulence of this strain is dependent on the genetic background of the rat. Specifically, we showed that *Toxo1*, a locus known to control the outcome of toxoplasmosis in rats infected with archetypal strains (Cavaillès et al., 2006), is involved in the modulation of the GUY008-ABE virulence.

The clinical signs described for the Guianese toxoplasmosis are pleiotropic including patients presenting pneumonia, cardiac or ophthalmological abnormalities. One patient was found to suffer of one organ failure while another died due to multiple organ failure (Demar et al., 2007, 2012). During the acute infection with GUY008-ABE parasites, F344 rats displayed signs of major general decrease in health. At day 14 post-infection, they were emaciated and underweight (20% loss). All rats also displayed orbital hemorrhage. Analysis of organ weights revealed, as in patients, hepato- and spleno-megaly. However, since both of these signs were also found in Prugniaud-infected F344 rats, these are likely more associated with *Toxoplasma* infection rather than to strain virulence. Only the lung weight was significantly higher in the GUY008-ABE-infected rats. Lung involvement was observed for all patients, with unilateral or bilateral crackles, unilateral bronchial breathing or areas of dullness (Demar et al., 2012). Thus, the lung weight increase is clearly related specifically to GUY008-ABE virulence.

Another major observation was the very high parasite load found in some organs. We have analyzed the parasite burden in organs known to be infected in the rat model (Zenner et al., 1998). In this work, the RH acute infection was monitored in F344 rats and low parasite numbers were found in the spleen and the lung at day 4 post-infection, and in the brain only at day 16 post-infection. Parasites were never detected in the heart and liver during the course of infection. On the contrary, here, we observed parasites in all tested organs and whatever the strain used. For the Prugniaud-infected rats, organ parasite loads were smaller than those observed with the GUY008-ABE strain and were almost stable between day 4 and day 14 post-infection. Results were different for the GUY008-ABE infection, organ parasite loads increased between day 4 and day 14 post-infection with a profile more closely aligned to the profile of parasite dissemination found in RH i.p. inoculated mice (Zenner et al., 1998). The RH strain is extremely virulent in mice as one parasite allows animal death within 10 days of infection (Howe and Sibley, 1995). At day 4 post-infection, mice present high parasite burden in all organs (10^5^ for the brain, 10^6^ for the heart, 10^7^ for the liver and 10^8^ for the spleen and the lungs) (Zenner et al., 1998). These numbers are close to those we found in the GUY008-ABE-infected rats especially for the heart and the lungs (10^5^ and 10^6^ parasites, respectively). Thus, the parasite loads found in the organs of GUY008-ABE-infected F344 rats were similar to that found in RH-infected OF-1 mice, and is likely to be associated with parasite hypervirulence.

The Guianese toxoplasmosis was associated with multiple organs failure. In the GUY008-ABE-infected F344 rats, lungs were found to be the most disrupted organ with histiocytic inflammation, alveoli full of serous fluid and presence of parasites foci. On the contrary, no tissue disruption was observed for the Prugniaud-infected rats. It has already been shown that the lung architecture can be modified by *Toxoplasma* infection in the rat (Foulet et al., 1994). Indeed, plurifocal fibrin alveolitis or acute bronchiolitis were observed in lungs of F344 nude rats after RH or Prugniaud infection, respectively (Foulet et al., 1994). Here, the GUY008-ABE acute infection was performed in the immunocompetent rat F344. Therefore, despite an active immune system, atypical strain led to similar lung failure, as in the case of RH infection of immunocompromised nude rat. Altogether, in GUY008-ABE-infected F344 rats, the observed physiopathology (loss of rat weight, lungs weight increase associated with high parasite burden and tissue destruction) suggests a severe respiratory failure leading to the death of animals and is fully concordant with the pathology observed in Guianese patients.

The severity of toxoplasmosis observed in the F344 rats infected with the atypical hypervirulent GUY008-ABE (LD100 = 10^7^) provides evidence that the lethal effect is due to the parasite genotype. Indeed the same route and inoculum of infection by Prugniaud parasites did not cause F344 rat mortality nor acute clinical signs. Up until now, *Toxoplasma* virulence and its association with parasite genotype have been widely investigated in the mouse model using the archetypal I, II and III clonal lineages (Saeij et al., 2006; Taylor et al., 2006). Their virulence in mice differs substantially, with the type I strain being acutely virulent (LD100: 1 to 10 viable organisms), type II strains exhibiting intermediate virulence (LD50 >10^3^ in inbred mice and >10^5^ in outbred mice), and type III being avirulent (Howe and Sibley, 1995; Sibley and Ajioka, 2008). However, compared to humans, mice are naturally more sensitive to *Toxoplasma* infection and recent studies have shown that they employ distinct innate pathways to control the infection (Gazzinelli et al., 2014). By contrast, in the F344 rats, the clinical course of infection between rats and humans is similar, with infection by the Prugniaud strain (type II) leading to an asymptomatic chronic infection (Darcy and Zenner, 1993; Zenner et al., 1998). Moreover, infection with the RH strain (type I) is totally controlled (not lethal) even with high inoculum (>10^8^ parasites) (unpublished data), supporting the notion that divergent mechanisms of resistance between rat and mice are at work (Gazzinelli et al., 2014). Hence, surprisingly, when inoculated in CD1 mice, the GUY008-ABE strain was responsible for only 83% of animal death at 30 days post-infection (Khan et al., 2014). However, the lack of any defined LD50 or LD100 in this study hampers valuable comparison.

In the rat model, the outcome of toxoplasmosis is directed by the *Toxo1* locus (Cavaillès et al., 2006; Cavailles et al., 2014). Indeed, all described refractory rat strains bear a highly conserved *Toxo1*-LEW haplotype while susceptible rats like the F344 or the BN strains bear divergent *Toxo1* haplotypes. Rats harboring the *Toxo1*-LEW haplotype block do not develop anti-*Toxoplasma* serology nor brain cysts when infected with Prugniaud or RH strains (Cavailles et al., 2014). Our data revealed that, unexpectedly, LEW rats infected by GUY008-ABE parasites developed positive serologies (not shown) and brain cysts. To our knowledge, this is the first time that a parasite strain is described as being able to bypass the LEW refractoriness *in vivo*, demonstrating that the *Toxo1-*control of *Toxoplasma* infectivity is dependent on the parasite genotype. However, even if *Toxo1* is not able to prevent GUY008-ABE infection, it modulated the parasite burden, since 20-fold reduction in the number of cysts was found in the brain of LEW rats compared to the susceptible LEW.BN-c10-F rats (BN *Toxo1*-haplotype). In rats of the *Toxo1*-LEW haplotype, the lack of serological response together with the undetectable local parasite burden (Sergent et al., 2005) indicated that parasite elimination is very efficient and occurs at the level of natural barriers. Moreover, using RH type I parasites, it has been shown that *Toxo1* controls very rapidly the local spreading of the parasite after i.p. infection (Cavaillès et al., 2006). It seems therefore that depending on the parasite strain, *Toxo1* could modulate the parasite infectivity and spreading.

Up to now, the *in vivo Toxo1*-mediated resistance has been strictly correlated to both the inhibition of parasite proliferation *in vitro* and the death induction of both macrophage and parasites (Cavailles et al., 2014). Similar observation was described in LEW bone marrow-derived macrophages (Cirelli et al., 2014). As the GUY008-ABE strain is able to bypass the LEW resistance *in vivo*, we expected to observe *in vitro* parasite proliferation and decrease of cell death in the peritoneal LEW macrophages. Since this was not observed, it appears that depending of the parasite genotype, the inhibition of parasite proliferation within the macrophages *in vitro*, does not strictly correlate with the rat resistance to infection *in vivo*. This suggests that the *Toxo1* resistance is a complex trait, which may involve other cells and mechanisms of innate immunity.

In conclusion, we have established that the F344 is a pertinent model in reproducing the pathophysiology of infected patients by hypervirulent *T. gondii* strains. We also provided evidence that virulence of *Toxoplasma* strains is highly modulated by the *Toxo1* haplotype. Therefore, the rat model opens new avenues to discover new host-parasite interacting genes involved in the virulence of *Toxoplasma gondii*.

## Ethics Statement

This study was carried out in accordance with the recommendations of Breeding and experimental procedures were carried out in accordance with national and international laws for laboratory animal welfare and experimentation (EEC Council Directive 2010/63/EU, September 2010). Experiments were performed under the supervision of C.L. (agreement 38 10 38) in the Plateforme de Haute Technologie Animale (PHTA) animal care facility (agreement C3851610006 delivered by the Direction Départementale de la Protection des Populations) and were approved by the ethics committee of the PHTA (ComEth) and by the French government (APAFIS#7617-2016111710364203 v3).

## Author Contributions

CL designed and performed experiments, wrote article. AM and SK performed *in vivo* experiments. VC did the histological part. JB did the histological analysis. PC wrote article. M-FC-D designed experiments and wrote article.

### Conflict of Interest Statement

The authors declare that the research was conducted in the absence of any commercial or financial relationships that could be construed as a potential conflict of interest.
